# Disclosure of Genotype Information to Reduce Caffeine Intake in Slow Metabolizers: Findings from a Randomized Controlled Trial on Personalized Dietary Interventions

**DOI:** 10.3390/nu17203236

**Published:** 2025-10-15

**Authors:** Ewa Bulczak, Agata Chmurzynska

**Affiliations:** Department of Human Nutrition and Dietetics, Poznań University of Life Sciences, 60-624 Poznań, Poland; ewa.bulczak@up.poznan.com

**Keywords:** personalized nutrition, nutrigenetics, *CYP1A2*, caffeine intake, EMA, mobile application, food intake assessment

## Abstract

**Background/Objectives:** This study evaluated whether personalized nutrition (PN) advice combined with disclosure of genetic information leads to a greater reduction in caffeine consumption than PN advice alone in slow caffeine metabolizers in the short and long terms. Additionally, Ecological Momentary Assessment (EMA) was considered for its potential to improve dietary intake assessment. **Methods**: In 2019–2021, 94 adults (aged 18–60 years, C allele carriers of rs762551 *CYP1A2*, consuming ≥ 200 mg/day caffeine), 63% of whom were women, participated in a twenty-week intervention. Participants were randomized to receive PN with genotype information (the intervention group, n = 55) or without it (the control group, n = 39). All participants were advised to limit caffeine intake to 100 mg/day. Caffeine intake was assessed using a food frequency questionnaire and a smartphones application. After three years caffeine intake was reassessed. **Results**: After the intervention, caffeine consumption decreased (intervention group: 380.69 ± 217.58 to 153.73 ± 98.19 mg/day; control group: 394.44 ± 256.29 to 169.87 ± 85.70 mg/day; *p* < 0.01), with no group differences (*p* = 0.41). Three years later, a reduction (*p* < 0.01) was still observed in the intervention group, but the effect of time x group was insignificant. In total, 63% of the intervention group and 51% of the control group responded to at least three EMA prompts per day for at least three days. **Conclusions**: PN seems to affect caffeine intake in the long term. However, including genotype information in PN is no more effective than receiving PN recommendations without genetic information. EMA’s effectiveness in large-scale nutritional research may be limited due to the relatively low response rate.

## 1. Introduction

Personalized nutrition (PN) is a recent trend in human nutrition that has garnered significant interest from both the public and scientists. Moving away from conventional one-size-fits-all approaches may pave the way to better population health, including a reduced burden of noncommunicable diseases [1]. Personalized dietary recommendations should be customized to individual needs, and thus a range of data can be taken into account. Beyond age, gender, and physiological status (pregnancy, lactation), which are accounted for in the norms [2], recommendations can include also nutritional status (including malnutrition, obesity), medical conditions (such as metabolic disease, eating disorders, allergies, and food intolerances), physical activity level (amateur sports, professional sports, sedentary lifestyle), or type of occupation (such as shift work) [3]. Genetic variation can also be considered in personalized dietary recommendations [1].

There are currently no established genotype-based PN recommendations, apart from the those regarding caffeine intake proposed by the International Society of Nutrigenetics & Nutrigenomics (ISNN) in 2016 [4]. These recommendations are based on the assumption that carriers of at least one C allele in the rs762551 *CYP1A2 * gene, which encodes a caffeine metabolizing enzyme, metabolize caffeine more slowly (classified as slow metabolizers), which leads to a prolonged physiological effect of caffeine and may increase the risk of adverse health outcomes associated with high caffeine intake, including a higher risk of nonfatal myocardial infarction (MI) [5]. Although caffeine intake may also contribute to insomnia, increased anxiety, gastrointestinal discomfort, tremors, and headache [6], we did not investigate these health effects. The ISNN recommends that slow metabolizers limit caffeine consumption to one cup per day in order to reduce these risks [4]. The caffeine content corresponding to this recommended one cup is approximately 100 mg on average [7], which is a quarter of the daily level set as safe by the European Food Safety Authority (EFSA)—namely, 400 mg caffeine [6].

Studies of the advantages of incorporating PN into dietary counseling have yielded contradictory results [8,9,10,11]. Benefits of disclosing genotype information in dietary counseling have been both observed [12,13,14,15,16], but have also been excluded [17,18,19,20,21]. Meta-analyses of the effects of informing individuals of their increased genetic risk of a disease on their motivation and ability to make changes in the intake of nutrients or foods have also proved inconclusive, as have meta-analyses of whether disclosing genetic information alone leads to desired changes in adults [22,23,24]. Considering the above, the effectiveness of PN still needs to be validated. The main goal of the present study was thus to assess whether PN advice, combined with the disclosure of genetic information, could result in a greater decrease in caffeine than receiving PN recommendations without genetic information. Specifically, our recommendations were focused on reduction in coffee intake in slow caffeine metabolizers. Moreover, long-lasting effects of PN were evaluated in a follow up study.

Due to the critical need for precise measurement of food intake in nutrigenomic studies [24], where phenotyping is a key element in a genotype–phenotype association study, a secondary objective of the present study was to evaluate the effectiveness of an application for mobile devices designed to measure caffeine intake. This application allows the collection of real-time data on participants’ states and behaviors using a smartphone—an approach referred to as Ecological Momentary Assessment (EMA). EMA involves repeated measurements of participants’ experiences and behaviors within their natural environment. including factors that follow specific events [25]. EMA is considered to be less prone to retrospective self-report biases because participants are answering questions about their present experience. Despite the promise of such technological advances and the strong interest in ecological models of health behavior, there have yet been few studies describing and validating usable tools that measure diet, physical activity, and the environment [26,27]. The present study is intended to contribute to filling this gap.

## 2. Materials and Methods

### 2.1. Study Design

Recruitment of people of both sexes aged 18–60 was carried out between November 2019 and June 2021 at the Department of Human Nutrition and Dietetics, Poznań University of Life Sciences, Poland. Advertisements were distributed in the national and local press, on radio, and on television. The snowball sampling method was then used, with participants being asked to further advertise the study among friends and family.

**Figure 1:**
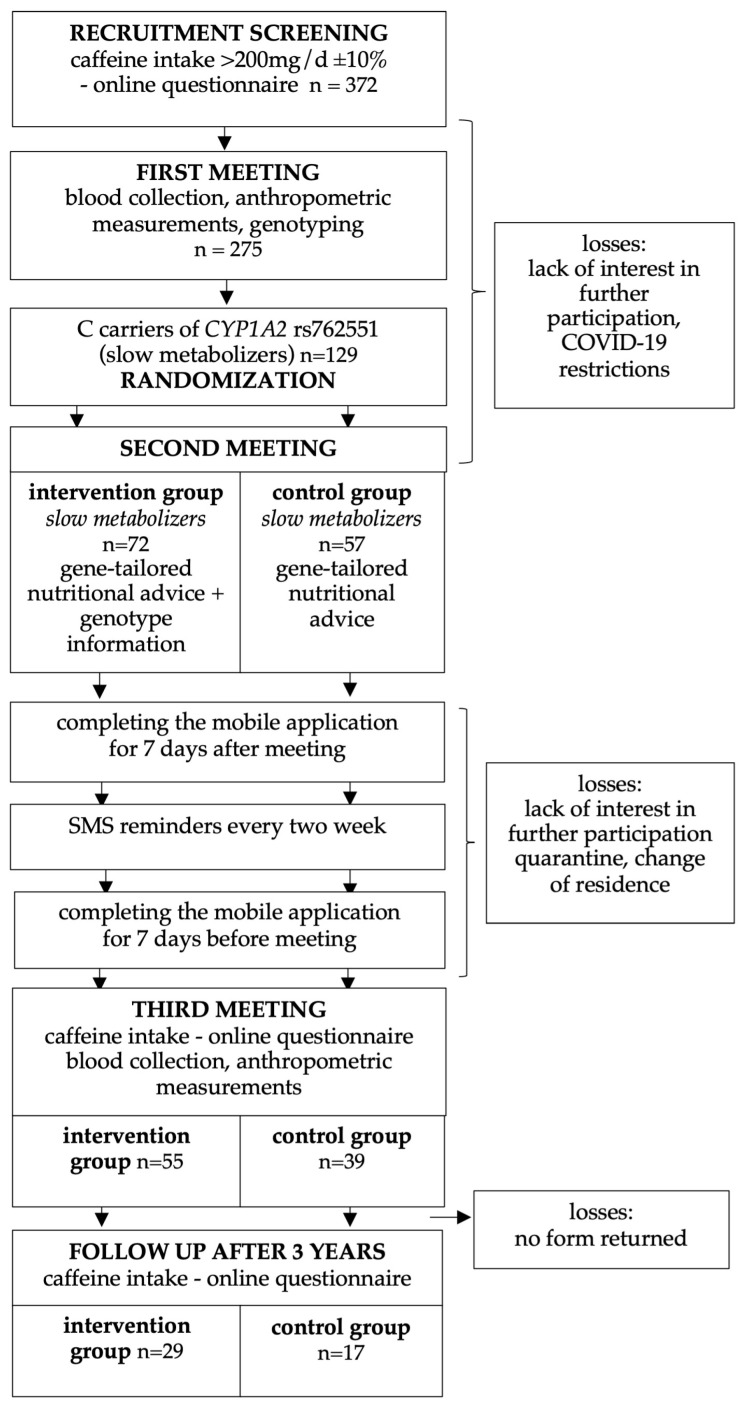
Study flowchart.

The required sample size, assuming a significance level (α) of 0.05, power (1–β): 0.80 and a moderate effect size, was 64 participants in both study groups. The screening group size was determined using the assumption that 50% of adults drink two or more cups of coffee per day, amounting to approximately 200 mg of caffeine [6,28], and that the frequency of the C allele of the rs762551 *CYP1A2* gene in the Caucasian population is around 30% (https://www.ncbi.nlm.nih.gov/snp/rs762551, accessed on 10 August 2022). Therefore, screening approximately six hundred individuals of both sexes, aged 18–60, for caffeine intake would yield about three hundred subjects to be further screened for the *CYP1A2* genotype. The attrition rate was initially calculated as 10%. However, due to the COVID-19 pandemic (with its associated lockdowns, quarantines, and changes in residence), the dropout rate was significantly higher than anticipated. More than six hundred people expressed interest in participating in the project, but only 372 people completed the on-line screening questionnaire. Of these, 275 attended the first meeting and were genotyped to identify C carriers of the rs762551 *CYP1A2* gene (classified as slow caffeine metabolizers), who were the target group of the study. The final study group consisted of 129 healthy *CYP1A2* C carriers living in the Poznań area with a daily caffeine intake of at least 200 ± 20 mg. These participants were randomized into an intervention (n = 72) and control (n = 57) groups, using an online tool (http://www.randomizer.org/). No restrictions were made on the randomization. Finally, 94 subjects (35 men and 59 women) completed the entire trial (Figure 1).

The inclusion criteria were: slow metabolizer (C allele carriers in allele in rs762551 CYP1A2), caffeine intake above 200 mg/d. The exclusion criteria were: being a rapid metabolizer (homozygotes AA allele in rs762551 CYP1A2), chronic diseases (e.g., cancer), pregnancy, breastfeeding, limited ability to communicate to the extent that it was impossible to provide nutritional advice, eating disorders (as determined during a nutritional interview), and injuries that prevented participants from eating their usual diet.

The trial was registered on ClinicalTrials.gov as number NCT04122053. The Bioethics Committee of Poznań University of Medical Sciences approved the study (number 196/19).

### 2.2. Intervention

The study was designed with three individual meetings and with an eight-week intervention, but due to the epidemiological situation, the intervention duration had to be extended to twenty weeks. At the first meeting, participants were asked about sociodemographic and behavioral data, anthropometrical measurements were taken, and blood samples were collected for later analysis. Body weight [kg] was measured using ac scale with an accuracy of 0.1 kg in subjects in a fasted state, and body height was measured to an accuracy of 0.01 m while standing without shoes, with the use of a stadiometer. Body mass index (BMI) was calculated as weight in kilograms divided by height in meters squared. In accordance with the WHO criteria BMI value higher than 25 kg/m^2^ was indicated as overweight and obese [29]. Waist, hip, arm and thigh circumferences were measured by trained staff. At the second meeting (held in person or online), each participant received advice to reduce their caffeine intake to below 100 mg per day. The intervention group also received information on their genotype and their increased risk of MI. Each participant received reminder text messages every two weeks for the whole intervention period (see Figure 1). Directly after the meeting, participants began using an app in which they assessed their caffeine intake for one week. After twenty weeks, participants were invited to a third meeting, where blood was collected again for biochemical testing and body composition analysis was performed. Prior to this meeting, participants had filled out an online caffeine intake questionnaire and had also completed assessments in their phone app for a full week. After the third meeting, all participants received a final report containing all data (anthropometry, biochemistry, and gene variant).

To evaluate the long-lasting effects of personalized recommendations, a follow-up study of caffeine intake was conducted three years later in the form of an online caffeine intake questionnaire.

### 2.3. Caffeine Intake Assessment

We used two methods to assess caffeine intake: a validated caffeine consumption questionnaire [30] and a smartphone app. Before filling out the questionnaire, each participant received information on how to complete it. Participants were asked on three occasions to provide their exact daily consumption of caffeinated products: when being screened for participation (at the beginning of the study), at the end of the study, and after three years (Figure 1). Additionally, all participants were requested to complete the assessment in the mobile application (authored by the company ITgenerator, Poznan, Poland) at the beginning and at the end of the study. This application was designed to record caffeine consumption in a real-time manner (EMA) and was based on the caffeine intake questionnaire. The application was installed on the participant’s phone or on a phone provided for them, and reminded them four times per day to input information on the caffeine-containing products they had consumed. In order to prevent duplicate entries, access to the application was blocked from the moment when answers were input until the next prompt occurred. In the analysis of the results, only individuals who gave replies to at least three prompts per day on at least three days in both weeks were included. The use of the application was abandoned in the follow up study, due to problems reaching participants.

### 2.4. Genotyping

DNA was isolated from a 200 µL blood sample to the manufacturer’s protocol (NucleoSpin Blood kit, Macherey-Nagel, Düren, Germany). DNA quality and concentration were determined using a microvolume spectrophotometer (DS-11, DeNovix, Wilmington, DE, USA). Genotyping of *rs762551* in *CYP1A2* gene was accomplished by real-time PCR using a TaqMan probe assay (Thermo Fisher Scientific SNP Genotyping Assay: C_8881221_40 for *rs762551* in *CYP1A2* gene, Waltham, MA, USA) and a LightCycler 480II (Roche Diagnostics, Basel, Switzerland), following the manufacturer’s protocol. The PCR reaction was performed in 10 μL of total volume, which included 1.5 µL of purified genomic DNA, 5.0 µL of LightCycler 480 Probes Master (Roche Diagnostics), 0.25 µL of SNP genotyping TaqMan probe assay (Thermo Fisher Scientific), and 3.25 µL of PCR-grade water (Roche Diagnostic).

### 2.5. Statistical Analysis

Statistical analysis was performed using Statistica 13.3 (StatSoft) with a significance level of α = 0.05. Descriptive statistics and contingency tables were used to determine the mean, standard deviation. and population size in the groups (control, intervention) and subgroups (women, men). The normality of the data distribution was assessed using the Shapiro–Wilk test. The assumption of equal variances was met, and the central limit theorem was used to compare all data with the parametric, paired and unpaired when appropriate, Student’s *t*-test [31]. Concurrently, in order to assess the independence of the two variables—*success* (defined as achieving the recommended caffeine intake level of 100 mg or less daily after the intervention) and *type* of study group—a chi-squared test with Yates’ correction was used, and Yule’s *Q*-correlation was additionally employed.

To assess changes in caffeine intake over time, a repeated-measures analysis of variance (ANOVA) was conducted with time (baseline, post-intervention, and three-year follow-up) as the within-subject factor and a study group (intervention vs. control) as the between-subject factor. Post hoc pairwise comparisons between time points were performed using Tukey’s HSD test with continuity correction. Effect sizes (partial η2) and observed power were reported to complement statistical significance.

## 3. Results

### 3.1. Characteristics of the Study Group

Ninety-four people aged 18–60 years successfully completed the entire intervention procedure (59 women and 35 men). The intervention group consisted of 18 men and 37 women, while the control group consisted of 17 men and 22 women. Table 1 shows baseline demographic and anthropometric characteristics for each group. The majority of participants were nonsmokers, who had completed higher education, who drank black unsweetened coffee and tea, and who were mostly employed full-time. The majority of participants slept seven or eight hours/day, and spent eight or more hours working at a computer. They generally described their nutritional knowledge as good, or in the case of 16%, as very good. The percentage of responses for each sociodemographic and behavioral aspect is shown in Supplementary Table S1. The C allele frequency of the *CYP1A2* gene (rs762551) was 0.31, while the frequency of the A allele was 0.69.

Follow-up data were compared only for people who completed the form, not for the entire population, meaning there are smaller numbers of people in both groups at follow-up: the intervention group consisted of 29 people (13 men and 16 women; mean age 34.14 ± 9.96 years) and the control group consisted of 17 (6 men and 11 women; mean age 36.24 ± 9 years).

### 3.2. Short-Term Changes in Caffeine Intake

#### 3.2.1. Short-Term Changes in Caffeine Intake Assessed by the Caffeine Intake Questionnaire Following the PN Intervention

The average caffeine intake in both groups decreased after the intervention by 59.62% in the intervention group and by 56.93% in the control group (Table 2). The decrease in consumption occurred in 52 out of 55 people (95%) in the intervention group and in 36 out of 39 people (92%) in the control group. The average change in total caffeine consumption (Δ) immediately after the intervention was not significantly greater in the intervention group (−211.13 ± 233.03 mg/day) than in the control group (−243.99 ± 301 mg/day) (*p* = 0.64).

Prior to the intervention, there had been no differences in caffeine intake between women and men, either in the entire study group (396.17 ± 272.98 mg/day vs. 370.03 ± 145.42 mg/day, *p* = 0.53) or within the separate study groups: 381.28 ± 251.01 mg/day vs. 379.71 ± 129.96 mg/day, *p* = 0.36 in the intervention group, 421.22 ± 311.01 vs. 359.78 ± 163.64, *p* = 0.92 in the control group. However, after the intervention, women consumed 22.05% more caffeine than men, but this difference was observed only in the entire study group (172.79 ± 93.2 mg/day vs. 134.69 ± 90.24 mg/day, *p* = 0.04) and in the intervention group (170.15 ± 94.14 mg/day vs. 110.45 ± 97.86 mg/day, *p* = 0.01) (Supplementary Table S2).

Caffeine consumption from different product groups did not differ between the control and intervention groups either before or after the intervention (Table 2). With the exception of yerba mate in the intervention group, caffeine consumption decreased in both groups after the intervention. In the intervention group, the percentage decrease in absolute values ranged from 47.65% for chocolate to 100%, for supplements, but yerba mate consumption increased by 945.9%. In the control group, the decrease ranged from 50% for chocolate to 100% for supplements, similar to those in the intervention group.

Achievement of the recommended intake level (≤100 mg) was independent of membership in the study group (Chi^2 Yates^ = 1.15; *df* = 1; *p* = 0.28), but looking at the results assessed with the Yule’s *Q*-correlation (Table 3), a small negative correlation Q = −0.30 can be seen: this may indicate that people would have tended to succeed if they had moved from the control group to the intervention group.

The majority of participants (69%) who achieved the recommended caffeine intake level were in the intervention group. However, in both groups, more than half of the participants did not meet the recommendation: these made up 67% of the intervention group and 79% of the control group (Table 3). It is of note that most participants (64% of the intervention group and 49% of the control group) reduced their intake by half, but there was no association between the assigned group and success in reducing by half caffeine intake (Chi^2Yates^ = 1.51; *df* = 1; *p* = 0.22).

#### 3.2.2. Short-Term Changes in Caffeine Intake Assessed by EMA Following the PN Intervention

Only 63% of respondents in the intervention group (n = 35) and 51% of respondents in the control group (n = 20) responded to at least three prompts per day for at least three days. The majority of the participants responded only twice a day and were therefore excluded from further analysis. The average caffeine intake at baseline did not differ between the two groups (146.70 ± 87.74 vs. 137.04 ± 102.33 mg/day, *p* = 0.71). After the intervention, caffeine consumption significantly decreased in both groups—by 59.1% in the intervention group and by 54.5% in the control group—but the differences between the groups were not statistically significant (82.71 ± 63.90 vs. 89.65 ± 43.69 mg/day, *p* = 0.67). It is of note that the statistically significant difference in caffeine intake before and after the intervention was observed only in the intervention group (*p* < 0.01.).

Similarly to the results obtained from the questionnaire, data from the application indicated that most participants who managed to conform to the recommended level of caffeine intake belonged to the intervention group (65%). However, in contrast to the questionnaire method, most participants in both groups achieved success in reducing caffeine consumption (60% of participants in each group). Reaching the advised level of caffeine intake and belonging to a particular group were independent (Chi^2^ = 0.82; *df* = 1; *p* = 0.77) (Table 3), and the same lack of correlation is indicated by Yule’s *Q* (Q = 0) (Table 3).

#### 3.2.3. Comparison of the Two Methods of Assessing Caffeine Consumption

Caffeine consumption was assessed in two ways: with the online questionnaire and using the EMA, and these methods showed a significant difference: After the intervention, intake in the intervention was 82.71 ± 63.90 mg/day vs. 142.32 ± 96.21 mg/day, n = 35; *p* < 0.01, while in the control group it was 89.65 ± 43.69 mg/day vs. 167.35 ± 71.14, n = 20; *p* < 0.01.

### 3.3. Long-Term Changes in Caffeine Intake: Three-Year Follow-Up Assessment Using the Caffeine Intake Questionnaire

More than half (53%) of the intervention group and nearly half (44%) the control group completed the three-year follow-up form. The intake they declared then showed there to be no significant differences in caffeine intake between the two groups. Although caffeine intake had increased over the immediate post-intervention levels, it did not return to baseline levels (Table 4). In both groups, caffeine intake after three years remained lower than the baseline intake (Δ = −40.27% in the intervention group and Δ = −36.60% in the control group), yet remained double the advised level.

Interestingly, significant differences between the three time points were observed in both groups (*p* < 0.01). However, Tukey’s post hoc analysis revealed a key distinction between the groups: in the intervention group, the differences in consumption between baseline vs. after intervention and baseline vs. follow-up were observed, but not in control group, indicating a clear trend of change over time in intervention group The caffeine intake in the study group three years after the intervention (227.94 ± 146.32 mg/day) was significantly lower than at baseline (381.63 ± 237.55 mg/day; *p* < 0.01). In the control group, the mean intake did not differ significantly between the two time points (follow-up 263.82 ± 136.92 mg/day vs. baseline 416.10 ± 290.88 mg/day; *p* = 0.06).

Repeated-measures ANOVA revealed a significant main effect of time, indicating that caffeine intake changed across the three measurement points (F(2, 88) = 29.61, *p* < 0.001). However, no significant main effect of the study group (F(1, 44) = 0.70, *p* = 0.41) or interaction between group and time (F(2, 88) = 0.02, *p* = 0.98) was observed (Table 4).

## 4. Discussion

We have demonstrated that, as a result of personalized dietary advice, a reduction in caffeine intake was achieved in over 90% of participants. Although we did not compare PN with standard dietary recommendations. However, contrary to our expectations, the observed changes were not greater in the group that received recommendations with genotype information than in the control group, which received recommendations without genotype information. Although in the long term a more pronounced decrease in caffeine consumption was observed only in the intervention group, the difference between the groups did not reach statistical significance.

These findings are challenging to discuss, as published studies on genotype-based PN remain limited. Consistent with our short-term results, previous research has shown that incorporating genetic information into dietary recommendations, or providing advice without giving such information, does not lead to a reduction in caffeine intake among individuals receiving genotype-based guidance. Mean changes in caffeine intake from baseline did not differ between the intervention and control groups at three and twelve months post-intervention [16]. It was notable in that study that the average baseline daily caffeine intake was lower than the advised intake level of 200 mg/day. In contrast, in our study, the initial caffeine intake in both groups significantly exceeded the level recommended for the slow caffeine metabolizers, although it remained within the safe consumption range defined by EFSA [6]. Furthermore, the advised caffeine intake level in our study was half the size (100 mg/day) of that in the previous intervention [16]. The difference between baseline caffeine consumption and the recommended level may be one reason for the substantial reductions in caffeine intake observed in our study: nearly 60% of participants in the intervention group and 55% in the control group achieved significant reductions. However, in neither study were different reductions in caffeine intake observed between the groups receiving different types of information [16]. This lack of difference could partly be explained by the strong motivation of the participants: some individuals underlined their desire to reduce caffeine consumption as a reason for participating in the study. It thus appears that merely indicating that it was necessary to reduce caffeine intake served as a sufficient stimulus for change, and that disclosure of genotype information did not change this situation much: it may have acted as a supplementary rather than decisive element in the short term. This means that being told that it was necessary to reduce caffeine intake and being informed of the health risks of not doing were processed within the framework of participants’ pre-existing cognitive schemas, facilitating their adherence to the recommendations [32,33].

Previous studies have suggested that the disclosure of genetic information as a part of PN can be unequally effective for different products and nutrients—being more effective for products that are perceived a priori by participants as undesirable in their diet (i.e., discretionary food and beverages, fat) [20,34,35,36,37]. This was also noted in our study: high levels of consumption of certain foods (chocolate, sweet beverages, and caffeine), were unwanted even at the beginning of the intervention. At the same time, other studies that analyzed the increased consumption of health-promoting products in groups receiving personalized recommendations noted changes only in a subset of the recommended food groups. The authors partially attributed this to the excessive number of suggested changes presented at once [38]. In the present study, we focused on caffeine. This makes the goal more attainable and is in line with idea that, for more effective dietary behavior changes, the goals set within the PN intervention should also align with the SMART criteria (“S” for specific, “M” for measurable, “A” for attainable, “R” for realistic, and “T” for timely). This may explain why changes in caffeine intake were observed directly after the intervention for both groups, but this was not enough to make the changes equally long lasting in both groups [38,39]. 

Although the outcomes are not entirely conclusive, some findings suggest that PN may have long-term benefits. A study with short-term results similar to ours, though focusing on the APOE gene, showed that genetic information did not immediately enhance the effectiveness of nutritional advice [17]. However, the changes made by participants who received this genetic feedback were still evident seven years later [18]. This was one of the reasons we decided to measure caffeine intake three years after the intervention. Although differences in intake between the two endpoints (immediately after the intervention and three years later) were statistically significant only in the intervention group (Table 4), there was no significant main effect of group or interaction between group and time (F(2, 88) = 0.02, *p* = 0.98). This indicates that the overall pattern of change was comparable across groups.

Our secondary outcome was related to our testing of the mobile phone application as a method for caffeine intake assessment. We hope that using the app would help improve this process, as obtaining reliable data is still a key challenge in nutrition studies [40]. Unexpectedly, the caffeine intake recorded in the app was 39% lower in the intervention group and 35% lower in the control group than the intake measured using the FFQ. This is inconsistent with results obtained by Chmurzynska et al., who observed an approximately 40% higher intake of high-fat products in the app than in the FFQ [27]. However, it seems characteristic of mobile app-based assessments that they record lower intake levels of energy and macronutrients (carbohydrates, fats, and less frequently protein) [41,42]. On one hand, this might suggest that using modern technologies for dietary assessment is more sensitive than standard methods. On the other hand, intentional underreporting by users should also be considered. Such observations have been presented by meta-analyses that noted that respondents intentionally underreported intake, particularly of discretionary foods, fat/sugar-rich beverages, and alcohol [42,43]. In our study, participants were asked to reduce caffeine intake, which could have led them to deliberately report lower consumption while using the app. However, it is important to note that they were instructed to list only those products they consumed every day when filling out the FFQ. It is therefore possible that not all caffeine-containing products were consumed with the daily frequency and for this reason they were not reported in the mobile app. 

As expected, participants demonstrated positive attitude towards the app [44]. Nevertheless, only a small percentage of participants (63% in the intervention group and 51% in the control group) responded at least three times, despite reminders and notifications. These results differ from those obtained by Chmurzynska et al. in a group of similar participants where high-fat food intake was assessed [27]. Low completion rate of EMA was also observed in an assessment of alcohol marketing exposure [45]. Daily response rates have also been noted to decrease with time: on average, completion rates decrease by approximately 2.0% per week [46,47]. The participants mostly used their own smartphones, with which they were very familiar. It has been hypothesized that participants tended to ignore well-known signals, and this has been suggested as another explanation of low response rates [46]. As mentioned above, many participants explained their lack of logging, especially at the end of the study, by claiming they were not consuming any caffeine-containing products. In such cases they did not submit as assessment on the app, despite reminder prompts beginning with the question, “Did you drink/eat anything since the last signal?”, to which “No” was one of the possible responses. Perhaps adding a message at the beginning of each prompt, stating that the app must be completed even if no caffeine-containing products were consumed, would increase the number of users completing the app. No such message was included in the application. Another cause of low responsiveness to the app’s signal might be the situation noted in the meta-analysis of Heron et al., who observed lower adherence to technology use among participants who had already learned and implemented the health behaviors promoted during the intervention using mobile devices (e.g., after eight to twelve weeks of intervention, the participants used mobile apps less frequently) [44].

Despite the convenience to respondents, the benefits of collecting data in real time and in the participants’ natural environment, and the ease of monitoring participants’ progress during the intervention [43,44,48], the lack of response to the app’s prompts limits the potential for use of this method in large-scale studies, and the high dropout rate requires recruitment of a much larger number of participants. We should also bear in mind that not all responses can be included in analysis. In the present study, only participants who responded to at least three prompts for both weeks were analyzed. This criterion was applied to make sure that we analyze only these responses, which reflect the real intake. Additionally, the amount of data generated in studies with more respondents complicates monitoring of the responses. This is not the case when paper forms are completed under researcher supervision.

## 5. Limitations

We are aware of some limitations of our study, and some have already been discussed. Apart from these, we did not consider the individuals’ attitudes to caffeine consumption, and this may have affected outcomes. Additionally, we performed the study during a pandemic, which could have influenced the caffeine consumption level. As we did not use the application in the follow-up study, it was not possible to compare both methods of assessing caffeine intake at all three time points, but this did not affect the effect of the intervention. The other limitation is that participants were randomized after the first meeting and genotype analysis, and slow metabolizers were invited to the second meeting. Despite numerous invitations, not all the randomized individuals were able to attend the second meeting, also due to COVID-19 restrictions. For this reason, the number of participants in each group is not equal. Despite that fact, there were no differences in any parameters at baseline. Another potential limitation is the possible impact of the COVID-19 pandemic on the study results. Firstly, the intervention period was extended from eight to 20 weeks due to epidemiological constraints. Secondly, some data were collected both before and after the outbreak of the pandemic, but our analyses did not reveal significant differences between these periods. Furthermore, data collected before the third meeting were obtained during the pandemic, ensuring that all participants were assessed under comparable conditions. For these reasons, we believe that the pandemic period did not significantly impact the study results. This aspect and its methodological implications were also discussed in a previous article [49]. Another issue is the accuracy and standardization of dietary intake assessment. As with all dietary assessment methods, none is entirely free from bias or error. Although the Food Frequency Questionnaire (FFQ) is a widely recognized and validated tool for estimating habitual nutrient intake, it also has well-known limitations, such as reliance on memory and self-reporting. To address this, we complemented the FFQ with EMA, which allowed real-time recording of dietary intake under participants’ actual living conditions. Nevertheless, the results obtained using the conventional caffeine consumption questionnaire and EMA differed substantially. Moreover, difficulties related to the use of the digital tool by participants revealed other challenges in accurately capturing habitual dietary intake. Nevertheless, both methods indicated the same effects of the intervention, supporting the robustness of our findings.

## 6. Conclusions

PN seems to affect caffeine intake in long term, but we did not compare PN with standard dietary recommendations. There were no significant differences in caffeine intake between the groups with and without genotype information, regardless of the assessment method applied. This suggests that disclosure of genotype information does not enhance compliance with PN advice. Moreover, EMA seems to offer a chance to collect data in a way that is convenient for participants, but its use in large-scale nutritional research seems limited due to the low response rate. These findings may help to fill the knowledge gap regarding implementation of the genotype-based dietary advice.

## Figures and Tables

**Table 1:** Group characteristics. Values are means ± standard deviations unless otherwise indicated.

Variable	Intervention Group (with Genotype Information) n = 55	Control Group (No Genotype Information) n = 39	*p* ^1^
age [year]	33.7 ± 10.6	34.2 ± 10.7	0.84
men/women [n/n]	18/37	17/22	0.29
smoking [%]	11.11	15.38	0.55
height [m]	1.79 ± 0.08	1.72 ± 0.09	0.33
body weight [kg]	71.18 ± 15.36	69.85 ± 15.17	0.68
BMI [kg/m^2^]	24.56 ± 4.6	23.45 ± 3.94	0.23
normal weight/overweight [n/n]	31/24	25/14	0.46
waist circumference [m]	0.84 ± 0.15	0.81 ± 0.13	0.30
hip circumference [m]	0.99 ± 0.09	0.96 ± 0.09	0.06

Abbreviations: BMI body mass index; Legend: ^1^ values were determined using the *t*-test to check for differences between the study and control group.

**Table 2:** Effects of PN intervention on intake of caffeine and caffeine-containing products, assessed using a caffeine intake questionnaire in a group of healthy people aged 18–60.

Food Product	Intake in the Intervention Group (with Genotype Information) n = 55						Intake in the Control Group (without Genotype Information) n = 39						*p* ^2^	*p* ^3^
	Before Intervention [mg/day]		After Intervention [mg/day]		*p* ^1^	∆	Before Intervention [mg/day]		After Intervention [mg/day]		*p* ^1^	∆		
	Mean	SD	Mean	SD		[%]	Mean	SD	Mean	SD		[%]		
caffeine total	380.69	217.58	153.73	98.19	**<0.01**	59.62	394.44	256.29	169.87	85.70	**<0.01**	56.93	0.78	0.41
Coffee ^4^	242.75	151.42	96.49	80.81	**<0.01**	60.25	257.70	208.92	118.66	68.50	**<0.01**	53.95	0.69	0.17
green and black tea	95.33	83.59	33.58	41.14	**<0.01**	64.77	84.01	74.88	40.92	40.18	**<0.01**	51.29	0.50	0.39
sweet beverages	12.48	33.13	5.80	27.44	0.25	53.53	5.66	11.63	1.45	5.09	**0.04**	74.38	0.22	0.33
energy drink	7.27	27.85	1.46	10.79	0.10	79.92	6.15	28.34	2.05	12.81	0.41	66.67	0.85	0.81
cocoa and chocolate	19.16	32.03	10.03	24.42	**<0.01**	47.65	12.96	13.89	6.48	8.86	**0.02**	50.00	0.26	0.39
supplements	3.11	17.46	0	0	0.29	100.00	24.90	78.28	0	0	**0.05**	100.00	0.05	-
yerba mate	0.61	3.19	6.38	40.26	0.68	−945.90	3.06	14.41	0.31	1.91	0.24	89.87	0.23	0.35
caf. mg/bm kg	5.50	3.13	2.25	1.54	**<0.01**	59.09	5.65	3.23	2.57	1.40	**<0.01**	54.51	0.96	0.18

Abbreviations: caf. mg/bm kg: caffeine intake in milligrams per kilogram of body mass; SD: standard deviation; Legend: ∆: percentage difference between baseline and post-intervention intake. ^1^ *p* values were determined using the paired *t*-test to check for differences before and after the intervention within groups, with bold values indicating statistical significance; ^2^ *p* values were determined using the *t*-test to check for differences between the study and control groups before the intervention. ^3^ *p* values were determined using the *t*-test to check for differences between the study and control groups after the intervention; ^4^ coffee: all types of coffee—ground, instant, etc.

**Table 3:** Distribution of success in achieving recommended caffeine intake (100 mg of caffeine or less) among participants in both groups.

Questionnaire				EMA			
Recommended Caffeine Intake	Control % in Column n = 39	Intervention % in Column n = 55	*p* ^1^ (Y)	Recommended Caffeine Intake	Control % in Column n = 20	Intervention % in Column n = 35	*p* ^1^ (Y)
Failure (% in row)	31 (46%) 79%	37 (67%) 54%	0.25 (−0.3)	Failure (% in row)	8 (36%) 40%	14 (64%) 40%	1.0 (0)
Success (% in row)	8 (31%) 21%	18 (69%) 33%		Success (% in row)	12 (35%) 60%	21 (65%) 60%	

Abbreviations: EMA, Ecological Momentary Assessment. Legend: ^1^ *p* values determined using Fisher’s two-way test; Y values determined using Yule’s Q-correlation to assess the independence of the two variables—*success* (defined as achieving the recommended caffeine intake level of 100 mg or less daily after the intervention) and *type* of the study group.

**Table 4:** Caffeine intake at three time points, data only for the follow-up participants.

Caffeine Intake [mg/day]	Intervention Group (with Genotype Information) n = 29		Control Group (Without Genotype Information) n = 17		*p* ^1^
	Mean	SD	Mean	SD	
baseline	381.63	237.55	416.10	290.88	0.66
after the intervention	127.20	89.39	150.22	68.28	0.36
follow up	227.94	146.32	263.82	136.92	0.42
**Comparison of caffeine intake in time (post hock)**	**Intervention group**		**Control group**		
	*p* ^2^		*p* ^2^		
baseline vs. after the intervention	**<0.01**		**<0.01**		
baseline vs. follow up	**<0.01**		0.06		
study group (1, 44) ^3^	η^2^ = 0.02; F = 0.70; *p* = 0.41				
time (2, 88) ^3^	**η^2^ = 0.40; F = 29.61; *p* < 0.01**				
time x study group (2, 88) ^3^	η^2^ < 0.01; F = 0.02; *p* = 0.98				

Abbreviations: SD: standard deviation. Legend: ^1^ *p* values were determined using the *t*-test to check for differences between the study and control groups; ^2^ *p* values post hoc Tukey; ^3^ time and study group effect repeated-measures ANOVA Friedman test with Kendall’s coefficient of concordance. Statistically significant values are presented in bold.

## Data Availability

The datasets used and analyzed in this study are available from the corresponding author upon reasonable request due to technical limitations.

## Supplementary Materials

The following supporting information can be downloaded at: https://www.mdpi.com/article/10.3390/nu17203236/s1, Table S1: Sociodemographic and behavioral data; Table S2: Caffeine intake before, twenty weeks after, and three years after the intervention aimed at decreasing caffeine intake in a group of healthy people aged 18–60; by sex.

## Abbreviations

The following abbreviations are used in this manuscript:

BMI	body mass index
caf. mg/bm kg	caffeine intake in milligram per kilogram of body mass
DM2	type 2 diabetes
EMA	ecological momentary assessment
ISNN	International Society of Nutrigenetics & Nutrigenomics
SD	standard deviation
