# Supplementary material for: Disclosure of Genotype Information to Reduce Caffeine Intake in Slow Metabolizers: Findings from a Randomized Controlled Trial on Personalized Dietary Interventions
Source: Nutrients. 2025 Oct 15;17(20):3236. doi: 10.3390/nu17203236 (PMC12567419; doi:10.3390/nu17203236)
Supplement: Supplementary file 1 [file nutrients-17-03236-s001.zip › nutrients-3906077-supplementary.pdf]

Supplementary Table S1: Sociodemographic and behavioral data

Category	Subcategory	% of answers
Education	vocational or secondary	24
	higher	76
Employment	no, I am retired or on disability pension	1
	no, I am on parental leave, unemployed, or a homemaker	1
	yes, but I work part-time	7
	yes, I am full-time employed	72
	I am a student	19
Hours slept on weekdays	6 or less hours a day	22
	7 or 8 hours a day	74
	9 or more hours a day	4
Hours slept on weekends	6 or less hours a day	3
	7 or 8 hours a day	68
	9 or more hours a day	29
How many hours a day do you spend on average watching TV or in front of a computer (including in work)?	less than 4 hours	21
	4 to almost 8 hours	37
	8 hours and more	42
Do you add sugar to coffee?	no	82
	yes	18
Type of coffee drank	black	48
	white (milk or cream)	52
Do you add sugar to tea?	no	82
	yes	18
Type of tea drank	black	70
	other (with milk, green, rooibos, pu erh, fruit, herbal)	30
Level of nutritional knowledge	insufficient	7
	sufficient	23
	good	53
	very good	18
Health status	worse than peers	6
	same as peers	62
	better than peers	32
How often do you dine out	never	5
	1–3 times a month	55
	once a week	29
	A few times a week	11

Supplementary Table S2: Caffeine intake before, twenty weeks after, and three years after the intervention aimed at decreasing caffeine intake in a group of healthy people aged 18–60; by sex

**Caffeine intake [mg/day]**	**Female**					**Male**				
	Intervention group (n = 37)		Control group (n = 22)		p_1	Intervention group (n = 37)		Control group (n = 22)		p^1
	mean	SD	mean	SD		mean	SD	mean	SD	
baseline	381.28	251.02	421.22	311.01	0.59	379.71	163.64	359.78	129.96	0.69
after intervention	170.15	94.14	177.23	93.63	0.78	110.45	75.94	160.35	97.86	0.10
follow up	238.68	163.61	268.23	118.75	0.61	214.73	177.94	255.72	127.09	0.57

[1] P values were determined using the *t*-test to check for differences within groups
